# Evaluation of a soluble CD14 subtype in patients with surgical sepsis

**DOI:** 10.1186/cc11694

**Published:** 2012-11-14

**Authors:** Y Fukui

**Affiliations:** 1 Kochi Health Sciences Center, Kochi, Japan

## Background

Surgical sepsis remains a cause of poor outcome. In order to treat surgical sepsis appropriately, it is important to reach diagnosis of sepsis early during the course and start antibiotic treatment. The gold standard of sepsis diagnosis is blood culture but there are issues with its sensitivity along with contamination. Soluble CD14 subtype (sCD14st) is an N-terminal fragment of CD14, and signal messenger of lipopolysaccharide (LPS). sCD14st is reported to increase in sepsis patients. In this study, we evaluated the specificity, clinical effectiveness of soluble sCD14st in patients with surgical sepsis.

## Methods

The study population was 18 operated patients in Kochi Health Sciences Center between April 2010 and March 2011. We investigated sCD14st using the compact automated enzyme immunoanalyzer PATHFAST (Mitsubishi Chemical Medience Co., Japan) and other biomarkers (procalcitonin (PCT), CRP, IL-6) at surgery (day 0) followed by 1, 3, 5, 7 days. APACHE II and Sequential Organ Failure Assessment (SOFA) were used to assess the sepsis severity. Sepsis was diagnosed according to the American College of Chest Physicians/Society of Critical Care Medicine (ACCP/SCCM) criteria.

## Results

There were nine patients with acute abdomen, esophageal cancer in five patients, cerebral bleeding in two patients, and a patient with Fournier's gangrene and trauma, respectively. The area under the curves (AUC) of receiver operating characteristic (ROC) curve analysis for septic infection were 0.92 for sCD14st, 0.82 for PCT, 074 for IL-6, and 0.82 for CRP. Two esophageal cancer patients who indicated over 800pg/ml in sCD14st had complicated nosocomial pneumonia. sCD14st also positively correlated with SOFA sore (*r *= 0.41) and APACHE II score (*r *= 0.28).

## Conclusion

sCD14st was a useful biomarker for the diagnosis of surgical sepsis and reflected clinical severity after operation.

